# Supplementary material for: Non-Abelian effects in dissipative photonic topological lattices
Source: Nat Commun. 2023 Mar 15;14:1440. doi: 10.1038/s41467-023-37065-z (PMC10017693; doi:10.1038/s41467-023-37065-z)
Supplement: Supplementary file 1 — Supplementary Information [file 41467_2023_37065_MOESM1_ESM.pdf]

Supplementary Information for "Non-Abelian effects in dissipative photonic topological lattices"

Midya Parto[1,*], Christian Leefmans[2,*], James Williams[1], Franco Nori[3,4,5], Alireza Marandi[1,2,†]

[1]Department of Electrical Engineering, California Institute of Technology, Pasadena, CA 91125, USA.

[2]Department of Applied Physics, California Institute of Technology, Pasadena, CA 91125, USA.

[3]Theoretical Quantum Physics Laboratory, RIKEN, Wakoshi, Saitama 351-0198, Japan.

[4]RIKEN Center for Quantum Computing, Wakoshi, Saitama 351-0198, Japan.

[5]Department of Physics, University of Michigan, Ann Arbor, Michigan 48109-1040, USA.

[*]These authors contributed equally

[†]marandi@caltech.edu

1 The effective Hamiltonian approach

In this section we derive effective Hamiltonians for the Lindblad master Eq. (1) in the main text corresponding to the different lattices implemented in our work. In general, one can write the effective non-Hermitian Hamiltonian as

$$\hat{H}_{\text{eff}} = \hat{H} - \frac{i}{2}\sum_j \hat{L}_j^\dagger \hat{L}_j. \tag{1}$$

We now consider several special cases for the dissipators $\hat{L}_j$. Here, we further assume that $\hat{H} = 0$. First, let us take $\hat{L}_j = \sqrt{\Gamma}(\hat{c}_j + \hat{c}_{j+1})$. By substituting in Eq. 1 one would obtain

$$\hat{H}_{\text{eff},1} = -\frac{i}{2}\Gamma\sum_j(\hat{c}_j + \hat{c}_{j+1})^\dagger(\hat{c}_j + \hat{c}_{j+1}) = -i\Gamma\sum_j \hat{c}_j^\dagger\hat{c}_j - \frac{i}{2}\Gamma\sum_j(\hat{c}_j^\dagger\hat{c}_{j+1} + \hat{c}_{j+1}^\dagger\hat{c}_j). \tag{2}$$

It is easy to verify that $\hat{H}_{\text{eff},1}$ corresponds to a non-Hermitian 1D lattice with uniform dissipative couplings between nearest-neighbor elements.

Next, we take the SSH model discussed in the manuscript with $\hat{L}_{A,j} = \sqrt{\Gamma_A}(\hat{c}_{A,j} + \hat{c}_{B,j})$ and $\hat{L}_{B,j} = \sqrt{\Gamma_B}(\hat{c}_{A,j+1} + \hat{c}_{B,j})$. The effective Hamiltonian for this system is given by

$$
\begin{aligned}
\hat{H}_{\text{eff},2} = &- \frac{i}{2}\sum_j \Gamma_A(\hat{c}_{A,j} + \hat{c}_{B,j})^\dagger(\hat{c}_{A,j} + \hat{c}_{B,j}) + \Gamma_B(\hat{c}_{A,j+1} + \hat{c}_{B,j})^\dagger(\hat{c}_{A,j+1} + \hat{c}_{B,j}) = \\
&- \frac{i}{2}(\Gamma_A + \Gamma_B) \sum_{x=A,B;j} \hat{c}^\dagger_{x,j}\hat{c}_{x,j} - \frac{i}{2}\Gamma_A \sum_j (\hat{c}^\dagger_{A,j}\hat{c}_{B,j} + \hat{c}^\dagger_{B,j}\hat{c}_{A,j}) \\
&- \frac{i}{2}\Gamma_B \sum_j (\hat{c}^\dagger_{B,j}\hat{c}_{A,j+1} + \hat{c}^\dagger_{A,j+1}\hat{c}_{B,j}).
\end{aligned}
\tag{3}
$$

The right-hand side of the Eq. 3 clearly describes a non-Hermitian SSH lattice with dissipative couplings Γ_A and Γ_B between successive lattice elements.

2 Relationship Between the Master Equation and Dissipative Delay Line Couplings

In this section, we discuss the relationship between the Lindblad master equation presented in the main text and the dissipative couplings utilized in our experiments. In Fig. 1, we compare the system modeled by the Lindblad master equation with a 1D chain of dissipatively coupled photonic resonators. As we discuss in Ref. (38), while the schematic in Fig. 1b considers spatial dissipatively coupled photonic resonators the delay lines in our time-multiplexed resonator network also constitute dissipative couplings. In fact, the delay lines act analogously to the bus waveguides in Fig. 1b, except that they couple time-multiplexed resonators rather than spatial resonators.

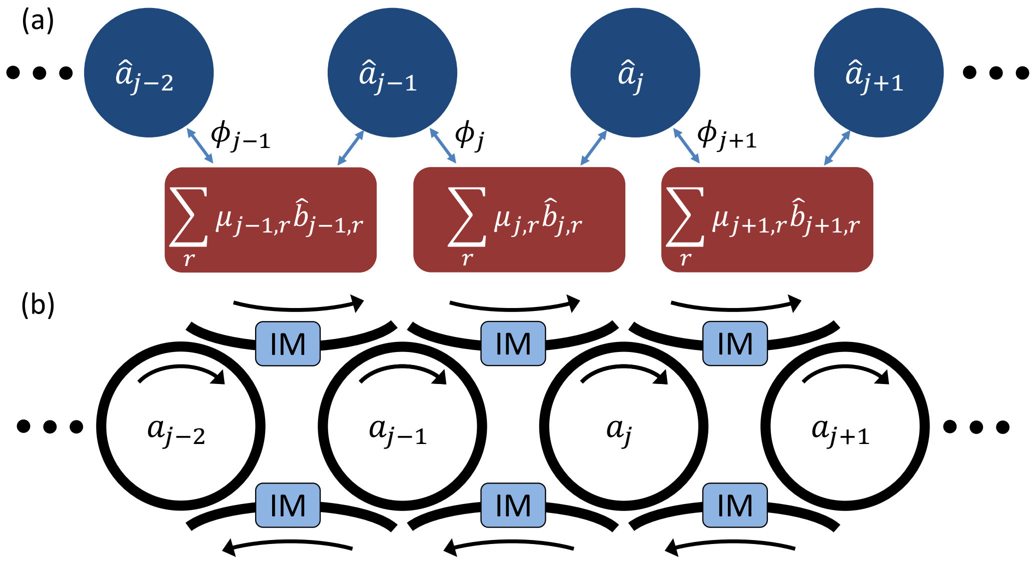

Figure 1: **Relationship Between Master Equation and Dissipatively Coupled Photonic Resonators a**, A 1D chain of oscillators coupled through intermediate reservoirs. We model this system with a Lindblad master equation. **b**, A 1D chain of dissipatively coupled photonic resonators. The IMs in the bus waveguides control the strengths of the couplings between the waveguides. In the text, we show that there is a limit in which dynamics of the mean field in **a** correspond to the dynamics of the coupled resonators in **b**.

A major difference between the dissipatively coupled ring resonators and the system modeled by the Lindblad master equation in Fig. 1a is that the system in Fig. 1a contains bidirectional reservoirs, while the bus waveguides in Fig. 1b are unidirectional. The unidirectionality of the bus waveguides facilitates implementing nonreciprocal couplings between the photonic resonators, while the bidirectionality of the reservoirs in Fig. 1a suggests that this system requires additional dynamics if it were to achieve nonreciprocal couplings (62). Despite the differences between the two systems in Fig. 1, here we show that, in the case of reciprocal couplings, the mean field dynamics of the master equation correspond to dynamics of the dissipatively coupled resonators studied in our experiments.

We begin our analysis by explicitly writing down the Hamiltonian that describes the system shown in Fig. 1a:

$$\hat{H} = \sum_j \omega_0 \hat{a}_j^\dagger \hat{a}_j + \sum_{j,r} \mu_{j,r} \hat{b}_{j,r}$$
$$+ \sum_j \left(\hat{a}_j^\dagger \left(\sum_m \mu_{j,m} \hat{b}_{j,m} \right) e^{i\phi_j} + \hat{a}_j^\dagger \left(\sum_n \mu_{j+1,n} \hat{b}_{j+1,n} \right) + \text{H.C.} \right). \quad (4)$$

Here the $\hat{a}_j$ are the system operators, and they are assumed to have identical resonance frequencies ω_0. The $\hat{b}_{j,r}$ are the reservoir operators. The $\mu_{j,r}$, which in general are complex, describe the coupling rates between the system and the reservoir. We explicitly add an additional coupling phase between some of the system-reservoir couplings. This coupling phase will allow us to tune the phase of the dissipative coupling between adjacent oscillators in the system.

From the Hamiltonian in Eq. 4, we can write down the master equation for the system in Fig. 1a. Following some standard assumptions and assuming that the reservoir is a vacuum field, we find that

$$\frac{\partial \hat{\rho}}{\partial t} = \sum_j \left(-i \left[\omega_0 \hat{a}_j^\dagger \hat{a}_j, \hat{\rho} \right] + \Gamma_j \left(\hat{L}_j \hat{\rho} \hat{L}_j^\dagger - \frac{1}{2} \left\{ \hat{L}_j^\dagger \hat{L}_j \hat{,} \rho \right\} \right) \right), \quad (5)$$

, where $\hat{L}_j = \left(\hat{a}_j + e^{-i\phi_j} \hat{a}_{j-1} \right)$ and where we have set $\hbar = 1$. The rates Γ_j, which are real-valued, depend on the coupling rates $\mu_{j,r}$ as well as the density of states in the $j\text{th}$ reservoir. We can use Eq. 5 to determine the equation of motion for the expectation value $\langle \hat{a} \rangle$. Noting that $\hat{a}$ is an operator in the Schrödinger picture, we find that

$$\partial_t \langle \hat{a}_j \rangle = \text{Tr} \left(\hat{a}_j \hat{\rho} \right) = \left(-i\omega_0 - \frac{\Gamma_j - \Gamma_{j+1}}{2} \right) \langle \hat{a}_j \rangle - \frac{\Gamma_j e^{-i\phi_j}}{2} \langle \hat{a}_{j-1} \rangle - \frac{\Gamma_{j+1} e^{-i\phi_{j+1}}}{2} \langle \hat{a}_{j+1} \rangle. \quad (6)$$

We simplify this expression further by making the assumption that $\phi_k = \pi \; \forall k$ and the substitution $\langle \hat{a}_j \rangle = A_j e^{-i\omega_0 t}$. Then we can write

$$\partial_t A_j = -\frac{\Gamma_j + \Gamma_{j+1}}{2} A_j + \Gamma_j A_{j-1} + \Gamma_{j+1} A_{j+1}. \quad (7)$$

Up to an additional term to model the intracavity losses, Eq. 7 is the equation of motion implemented by our time-multiplexed network when the delay lines are locked constructively to the main cavity (38). In Fig. 1b, the IMs control the coupling rates Γ_j in this equation. In the quantum system presented in Fig. 1a, this could be accomplished by either tuning the coupling rates to the reservoir or the density of states within the reservoir. By engineering these couplings so that $\Gamma_k = \Gamma_A$ if $k \bmod 2 = 0$ and $\Gamma_k = \Gamma_B$ if $k \bmod 2 = 1$, we find that the couplings in Eq. 7 reduce to those of the SSH model.

3 Mapping the Bloch Oscillation Hamiltonian to a Time-Multiplexed Photonic Resonator Network

There are several distinctions between our network and the familiar, continuous-time description of Bloch oscillations. Most saliently, the couplings of our network occur at discrete intervals and the PM responsible for emulating a linear potential implements a discrete phase ramp. Nonetheless, the dynamics of our network approximates the dynamics of Bloch oscillations. In this section we derive the mapping between the Bloch oscillation Hamiltonian and our setup.

We begin with the effective Hamiltonian that describes dissipatively coupled Bloch oscillations. We may express this Hamiltonian as

$$H = H_{\mathrm{C}} + H_{\mathrm{BO}} = \sum_n \{-iJ|n+1\rangle\langle n| - ij|n\rangle\langle n+1| + i\gamma|n\rangle\langle n|\} + \sum_n \alpha n|n\rangle\langle n|. \quad (8)$$

The equation of motion of this effective Hamiltonian may be written as

$$\partial_t|\psi\rangle = i\left(H_{\mathrm{C}} + H_{\mathrm{BO}}\right)|\psi\rangle. \quad (9)$$

This equation can be integrated formally to arrive at an expression for the time-evolution of $|\psi\rangle$:

$$|\psi(t)\rangle = \exp\left[i\left(H_{\mathrm{C}} + H_{\mathrm{BO}}\right)t\right]. \tag{10}$$

In the limit of a small time step, Δt, we can factor the exponential in Eq. 10 using the Baker-Campbell-Hausdorff formula. This yields an approximation with error on the order of $(\Delta t)^2$:

$$|\psi(t+\Delta t)\rangle = \exp\left[H_{\mathrm{C}}\Delta t\right]\exp\left[H_{\mathrm{BO}}\Delta t\right]|\psi(t)\rangle \tag{11}$$

We then make a second approximation by expanding the exponential term involving H_C. This approximation also introduces error of order $(\Delta t)^2$:

$$|\psi(t+\Delta t)\rangle = \left(\mathbf{1} + H_{\mathrm{C}}\Delta t\right)\exp\left[iH_{\mathrm{BO}}\Delta t\right]|\psi(t)\rangle. \tag{12}$$

This is the equation of motion implemented by our time-multiplexed network. To see why, let us consider the exponential term and the terms in parentheses separately.

First, we consider the exponential term, which is explicitly given by

$$\exp\left[iH_{\mathrm{BO}}\Delta t\right] = \exp\left[i\sum_n \alpha\Delta t n|n\rangle\langle n|\right]. \tag{13}$$

We can decompose the state vector $|\psi(t)\rangle$ in the position basis by writing it as $|\psi(t)\rangle = \sum_m c_m(t)|m\rangle$. Then, operating on $|\psi(t)\rangle$ with the exponential term gives

$$\begin{aligned}\exp\left[i\sum_n \alpha\Delta t n|n\rangle\langle n|\right]|\psi(t)\rangle &= \exp\left[i\sum_n \alpha\Delta t n|n\rangle\langle n|\right]\sum_m c_m(t)|m\rangle \\ &= \sum_m \exp\left[i\alpha\Delta t m\right]c_m(t)|m\rangle.\end{aligned} \tag{14}$$

Therefore, we see that the exponential term is equivalent to multiplying the sites of the state $|\psi(t)\rangle$ by a linearly increasing phase ramp, whose slope is set by $\Delta t\alpha$. In our time-multiplexed

network, in which the sites of our synthetic lattice are the pulses, we implement this phase ramp with our intracavity phase modulator.

To completely realize the dynamics described in Eq. 12, we must also implement the term in parentheses. This is accomplished by the combination of the delay line couplings and the feedback of the main cavity, which allows the state in the network to propagate from one roundtrip to the next. To see this, we can expand the term in parentheses as follows:

$$(\mathbf{1} + i\Delta t H_{\mathrm{C}}) = (1 - \Delta t\gamma)\,|n\rangle\langle n| + J|n+1\rangle\langle n| + J|n\rangle\langle n+1| \tag{15}$$

The first term on the right describes the propagation from one roundtrip to the next, while the second two terms describe the nearest-neighbor couplings between the pulses.

In summary, the sequential operation of the linear phase ramp followed by the implementation of the delay lines and the cavity feedback enables us to implement the dynamics described by Eq. 12 on each roundtrip of our time-multiplexed network. For this reason, our network implements the Hamiltonian Eq. 8 with an error $(\Delta t)^2$, where the time interval Δt is dictated by the $\sim 256\ \mathrm{ns}$ roundtrip time of our cavity. While the presence of this error prevents us from exactly implementing Eq. 8 with our time-multiplexed setup, this error is negligible for the network used in this work. This is evident from the correspondence between our measured values and the theoretical values predicted by the exact Hamiltonian.

4 Experimental Setup

To perform the Zak phase and Bloch oscillation measurements discussed in the main text, we used the setup shown in Fig. 2. This setup is design to have substantially lower loss than the time-multiplexed network employed previously in Ref. (38), and this enables us to observe more Bloch periods during our Bloch oscillation measurements. In this section, we walk through

the time-multiplexed network in Fig. 2, explaining the function of each optical and electronic component as we go along

Starting at the mode-locked laser in Fig. 2, the laser emits femtosecond laser pulses centered at $1550\ \mathrm{nm}$ with a $T_{\mathrm{R}} \approx 4\ \mathrm{ns}$ repetition rate. These pulses are sent through a $2\ \mathrm{nm}$ filter, which broadens them to $\sim 5\ \mathrm{ps}$. The broadened pulses are then split at a 90:10 splitter, which sends 10% of the incoming light directly to a $600\ \mathrm{MHz}$ detector. The output of this detector is passed through a $270\ \mathrm{MHz}$ low-pass filter, which isolates the fundamental frequency of the detector output. The output of the low-pass filter is used to time the FPGA that controls the electro-optic modulators (EOMs) in our experiments.

Meanwhile, the 90 port of this 90:10 splitter passes on to a 50:50 splitter. Half of the light is transmitted to an optical hybrid coupler, where it serves as a reference for measuring the in-phase and quadrature components of the pulses in our experiments. The other half of the light passes through two intensity modulators (IMs) before arriving at the input of our optical network. The first of these two intensity modulators is used to excite a particular state in the optical network. While all of the states excited in this work have uniform amplitude, we use IM_{00} to introduce a π phase shift between consecutive pulses. As in Ref. (43), a second IM, IM_{01}, is used to achieve a greater extinction ratio when we wish to stop injecting pulses into the network.

Light passing through the two IMs enters the optical network through another 90:10 splitter, which admits 10% of this light into the network and recirculates 90% of the light already present in the network. The light that enters the network enters directly into the "main cavity," which is the primary resonator of our optical system. The main cavity of this work supports 64 resonant pulses separated by the pulse repetition period, T_{R}. After leaving the 90:10 splitter, the light in the main cavity is split at another 50:50 splitter, which reserves half of the light in the main cavity and passes the other half into the optical delay lines. In Fig. 2, the delay line common

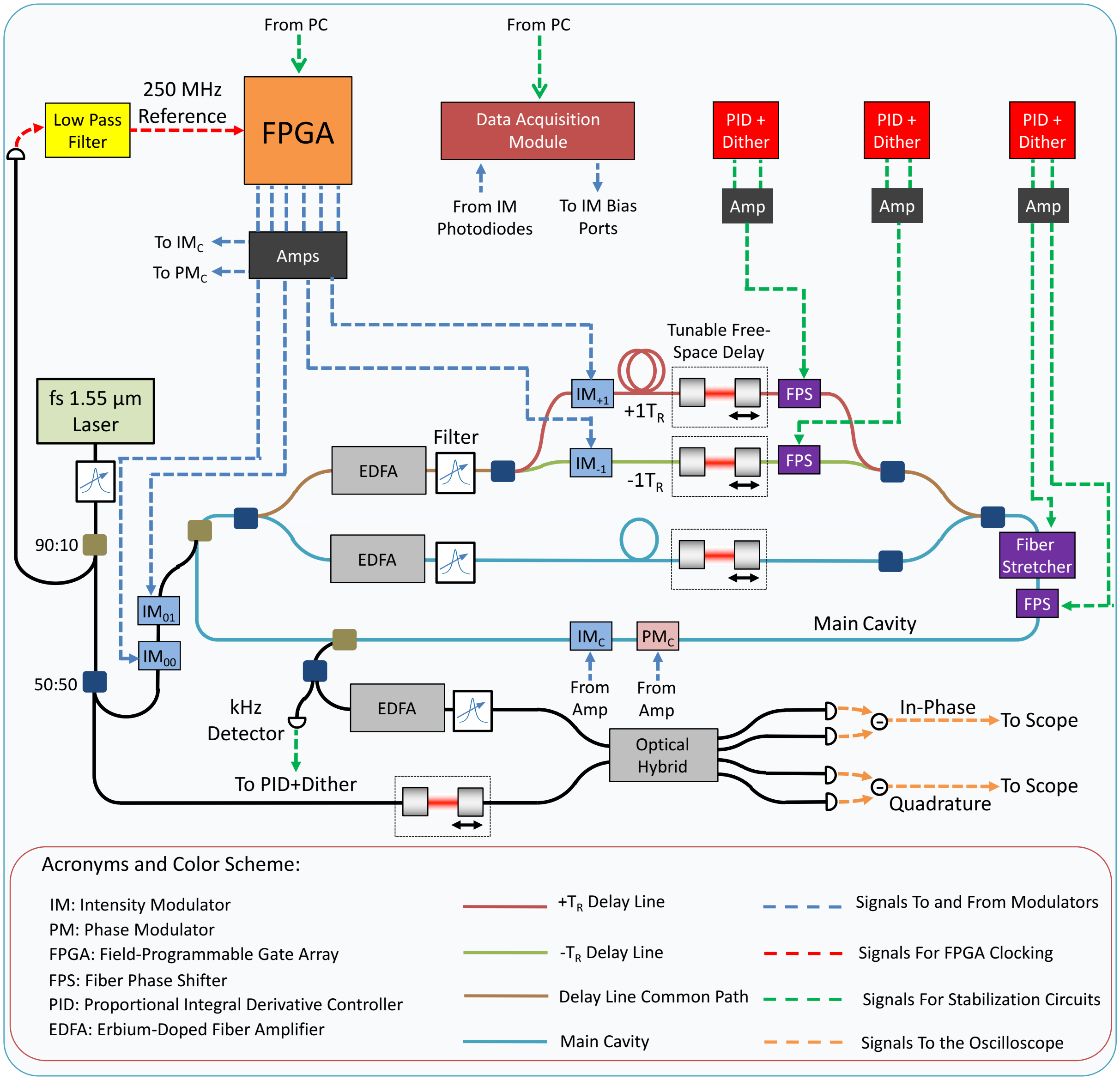

Figure 2: **A schematic of the experimental setup used in this work.**

path is colored brown, while the main cavity is colored blue.

After the 50:50 splitter, we insert EDFAs and $2\ \mathrm{nm}$ filters into both the delay line common path and into the main cavity. Placing EDFAs in both paths enables us to independently control the gain in the main cavity and the coupling strength in the delay lines. After the EDFA and filter in the delay line common path, we divide the light between the two delay lines at another 50:50 splitter. We choose the lengths of these two delay lines to couple nearest-neighbor pulses in the main cavity by delaying or advancing the pulses by one repetition period, T_{R}. The two delay lines are therefore referred to as the $+T_{\mathrm{R}}$ and $-T_{\mathrm{R}}$ delay lines, respectively. In both of the delay lines we insert an IM, a tunable free-space delay, and a fiber phase shifter (FPS). The tunable free-space delays are used to precisely set the lengths of the delay lines, while the fiber phase shifters are used for active stabilization. We drive the IMs with RF waveforms to modulate the couplings produced by the delay lines.

After these elements, the delay lines are recombined with each other at a 50:50 splitter and then recombined with the main cavity at another 50:50 splitter. This final 50:50 splitter is where light in the delay lines couples to pulses in the main cavity. The remainder of the main cavity consists of a fiber stretcher, an FPS, a phase modulator (PM), and an IM. Both the fiber stretcher and the FPS are used for active stabilization; a dither signal is sent to the FPS while a feedback signal is sent to the fiber stretcher, which has a longer dynamic range. As discussed in the main text, and as we discussed in Supplementary Section 3, the PM is used to approximate the linear potential responsible for Bloch oscillations. The IM is used to Q-switch the cavity, allowing us to briefly increase the gain seen by the pulses in the network while still remaining sufficiently below threshold.

After passing through these elements, a 90:10 splitter taps 10% of the light from the main cavity for detection while sending 90% of the light back to the intial 90:10 splitter into which light is injected. The light tapped from the main cavity passes through another 50:50 splitter.

One arm of the 50:50 splitter is sent to a slow, kHz detector, whose output is used as a locking signal for the main cavity and both delay lines. The other arm of the 50:50 splitter passes through another EDFA and $2\ \mathrm{nm}$ filter before being sent to an optical hybrid coupler. We choose to amplify our signal before the optical hybrid coupler rather than our reference because it is easier to adjust the length of the reference arm's tunable free-space delay to realize interference in the optical hybrid coupler if the signal power is stronger.

Our optical hybrid coupler contains two pairs of outputs, which we pass to balanced detectors to extract the in-phase and quadrature components of the pulses in the network. Note that in the case of our Bloch oscillation experiments, in which we are only interested in the intensities of the pulses, we pass the output of the cavity directly to a $5\ \mathrm{GHz}$ detector after the EDFA and filter rather than using the optical hybrid coupler.

In addition to the optical components, there are several electronic components that are worth mentioning. As briefly discussed before, the FPGA in Fig. 2 generates the RF driving waveforms for the modulators in our setup and is essential to controlling the state excited in the network, the couplings in the delay lines, and the linear potential responsible for Bloch oscillations. The FPGA outputs its RF signals to a series of amplifiers, which then transmit the signals directly to the modulators.

In addition to driving the modulators with RF signals, we also control the biases of the modulators with a dedicated data acquisition module (National Instruments).

Finally, we stabilize the network with a series of dedicated lock-in systems (Red Pitaya STEMLabs), which perform top-of-fringe dither locking using the FPSs and the fiber stretcher in the main cavity. Note that in the delay lines, the dither and feedback signals are summed together and both applied to the same FPS.

5 Calibration Procedure

In this section, we describe the procedures used to calibrate the intracavity PM and the IMs in the $\pm 1T_{\mathrm{R}}$ delay lines. These calibrations are used to generate the waveforms that implement the linear phase ramp within the main cavity and that produce the staggered site-to-site couplings of the SSH model.

Before jumping into these procedures, recall that our network supports 64 pulses. While calibrating these modulators, we use the first 32 pulses to establish a reference phase, and we modulate the phases of the remaining 32 pulses. This approach mirrors that used in our experiment (see Methods), where we also use the first 32 pulses in our cavity as reference pulses.

Moreover, we drive the modulators in our network with AC-coupled RF pulses, which are positive for $2\ \mathrm{ns}$ and negative for $2\ \mathrm{ns}$. Because the pulses are separated by $4\ \mathrm{ns}$, these RF pulses enable us to individually address each optical pulse as it passes through a given modulator. Because the duration of each pulse is only $\sim 5\ \mathrm{ps}$, we can tune the timings of the driving RF pulse sequences so that the optical pulses effectively see a constant modulation across the duration of the pulse. When we speak of implementing a phase ramp with our intracavity phase modulator, we mean that we sweep the amplitudes of the applied RF pulses so that we linearly sweep the phase modulation experienced by the pulses in our network.

We calibrate the intracavity PM with a two-step process. For the first step in the intracavity PM calibration, we inject 64 uniform pulses into our network and linearly sweep the RF voltage applied to the PM for the final 32 pulses. We measure the phase response with our optical hybrid and construct a plot of the measured optical phase versus the applied RF voltage. We use this mapping to generate a preliminary waveform for the linear phase ramp implemented during the experiment.

In the second step of our calibration, we fine tune our preliminary waveform to correct imperfections in our initial calibration. We perform this second step iteratively: We again inject 64 pulses into our network, and we apply the phase ramp waveform to the final 32 pulses. We measure the phase response of the pulses with our optical hybrid. We analyze the measured phases and compare them to the desired phase ramp. We then manually update our waveform to improve the accuracy of the implemented phases.

To calibrate the responses of our delay line IMs, we open the main cavity between the output and input 90:10 splitters so that there is no feedback in the cavity. We block the main cavity and one of the delay lines so that we only look at the transmission through one of the delay lines with our optical hybrid. Then, we inject 150 pulses into our network. For the first 50 pulses, we apply a constant amplitude drive, while for the next 100 pulses, we linearly sweep the drive from negative to positive voltages. We use the first 50 pulses to extract a phase reference, and we use the phase reference to generate an optical field versus applied voltage curve. From these curves, we generate preliminary waveforms for the couplings of the SSH model. We test these waveforms by sending a constant stream of pulses through the delay lines and by observing the throughput of the delay lines on a detector. If the throughput of a delay line does not correspond to the desired SSH couplings, we manually updated the delay line's driving waveform to improve the accuracy of the response.

6 Conservative and Dissipative Couplings

In this section, we review the differences between dissipative and conservative couplings, and we discuss why the delay lines in our time-multiplexed resonator networks implement the dissipative couplings described by the jump operators discussed in Section. 1. For a more detailed discussion on conservative and dissipative coupling, please see the Supplementary Information of Ref. (38).

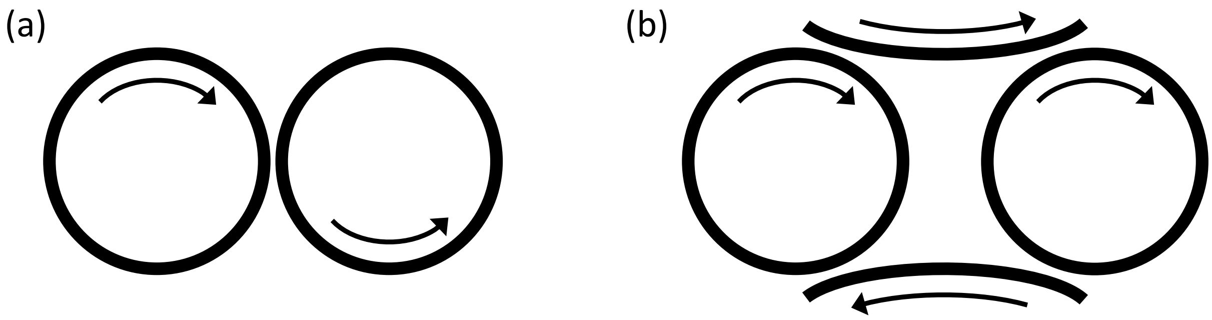

Figure 3: **Dissipative and Conservative Coupling.** **a**, A pair of conservatively coupled ring resonators. **b**, A pair of dissipatively coupled ring resonators.

We illustrate the differences between conservatively and dissipatively coupled resonators in Fig. 3. In Fig. 3a, we depict two conservatively coupled ring resonators. These resonators are conservatively coupled because they exchange information directly through their overlapping modes. This procedure conserves energy, and the conservation of energy imposes a strict set of phase relationships on the coupling between these two resonators. In mathematical terms, such conservative couplings are described by the Hamiltonian terms in Section. 1.

Figure 3b shows two dissipatively coupled resonators. In contrast to the conservatively coupled resonators, these dissipatively coupled resonators are coupled through bus waveguides, which act as intermediate reservoirs that join the two resonators. Energy is not conserved in this system, as light can exit through the open ports in the bus waveguides. The absence of energy conservation provides additional flexibility in terms of the coupling phases that we can achieve with dissipative couplings, as we can now tune the coupling phases simply by modifying the lengths of the buses. In mathematical terms, these dissipative couplings are described by the jump operators in Section. 1, which describe the process of coupling through an intermediate reservoir.

In our time-multiplexed resonator networks, the delay lines play the role of the bus waveguides in Fig. 3b. The splitter taps light from the pulses in the main cavity and transmits it through

the delay lines, and the combiner couples the light back into other pulses in the network. The phase accumulated and the attenuation experienced in the delay line ultimately determine the phase and strength of each coupling, which is essentially the parameter Γ in Eq. 2. However, while the parameter Γ is constant in Eq. 2, in practice, we can use the delay line IMs to vary the coupling strengths from pulse-to-pulse. Indeed, this is what we do to achieve the couplings of the SSH model in our network, which allows us to implement the staggered coupling strengths Γ_{A} and Γ_{B} in Eq. 3.

7 Dissipative discrete diffraction vs conservative

In this section we derive the analytical solutions pertaining dissipative discrete diffractions presented in Fig. 2b of the main manuscript. Consider the set of coupled equations

$$\frac{\partial}{\partial t}a_n = -2\kappa a_n + \kappa a_{n-1} + \kappa a_{n+1}, \tag{16}$$

subject to initial conditions $a_0(t=0) = A$ and $a_n(t=0) = 0$ for $n \neq 0$. From here, it is easy to see that the wave amplitudes can be obtained in terms of modified Bessel functions of the first kind $a_n(t) = I_n(2\kappa t)\exp(-2\kappa t)$. Figure 4 shows analytical results along with experimental measurements presented in Fig. 2b of the manuscript. As evident in this figure, dissipative discrete diffractions are qualitatively different from the conservative ones (50). More specifically, in the presence of dispersive couplings, which are represented by imaginary coupling coefficients in the system Hamiltonian, the interferences among different lattice sites oscillate between constructive and destructive. This leads to the familiar nulls in the diffraction patterns observed in such systems. Mathematically, this is described by the zeros of the Bessel functions of the first kind $J_n(x)$ which form the analytical solutions of the wave amplitudes in these lattices. The situation changes drastically when the lattice involves dissipative couplings which are represented by real coupling coefficients in the evolution equations Eq.16. Here, the analytical

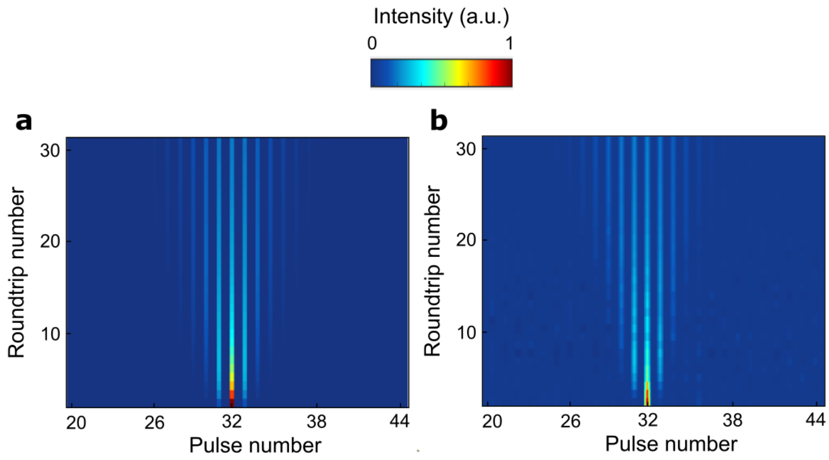

Figure 4: **Dissipative discrete diffractions. a,** Analytical results predicting the spread of light in a dissipatively coupled uniform lattice when one single site is intially excited. **b,** Experimental measurements obtained from the time-multiplexed setup corresponding to the scenario (**a**), as presented in Fig. 2b of the manuscript. Note that here, because of dissipative interactions, light amplitudes within different sites do not exhibit oscillatory behavior. In all cases, the optical power across the lattice sites is normalized in every round trip to provide a more distinct visualization of the field intensities.

solutions are instead given by the modified Bessel functions of the first kind $I_n(x)$ multiplied by exponentially decaying factors which only have nulls at the origin. In other words, here, the interferences among different sites are always in-phase and therefore do not lead to intensity nulls.

8 Dissipative Bloch Oscillations and topological invariants

Here, we formally show that by superimposing a linearly increasing potential over a 1D lattice with dissipative couplings that features a single dissipative band, one can achieve Bloch oscillations. We further show that by measuring the relative phases between the original field amplitudes and those after a full Bloch period one could directly measure the Berry phase associated with the corresponding 1D Hamiltonian.

Let us start with the dissipative evolution equation

$$\frac{\partial}{\partial t}|\psi(x,t)\rangle = \hat{H}_{\text{eff},0}|\psi(x,t)\rangle\,, \tag{17}$$

which supports Bloch eigenstates $|\psi(x,t)\rangle = \exp(ikx)\exp(-E(k)t)\,|u_k(x)\rangle$, where k is the Bloch momentum while $E(k)$ is a real Bloch eigenvalue that corresponds to the eigenstate amplification/decay in time. In addition, $|u_k(x)\rangle$ is the associated Bloch eigenstate within a unit cell characterized by the periodic boundary condition $|u_k(x+a)\rangle = |u_k(x)\rangle$, with a being the size of a unit cell taken to be equal to $a = 1$ here without any loss of generality. Next, we consider a linearly increasing potential term in the evolution equation

$$\frac{\partial}{\partial t}|\psi(x,t)\rangle = \hat{H}_t|\psi(x,t)\rangle\,, \hat{H}_t = \hat{H}_{\text{eff},0} + \hat{H}_1 \tag{18}$$

where $\hat{H}_1 = -i\sum_n \alpha n \hat{a}_n^\dagger \hat{a}_n$ and α is the gradient of the potential which is proportional to the magnitude of the effective force. Under these conditions, one can verify that if the system starts from an initial Bloch eigenstate denoted by $|\psi(x,0)\rangle = \exp(ik_0x)\,|u_{k_0}(x)\rangle$, then after a time evolution according to Eq. 18 it ends in the state $|\psi(x,t)\rangle = \exp[-\gamma(t)]\exp[i\phi(t)]\exp[i(k_0 - vt)x]\,|u_{k_0-vt}(x)\rangle$, which equals to the Bloch eigenstate at $k = k_0 - vt$ multiplied by a phase and amplitude factor. In this case, it can be verified that $v = \alpha/a = \alpha$ and

$$\begin{aligned} \gamma(T_B) - \gamma(0) &= \frac{1}{v}\int_{k_0}^{k_0-2\pi} E(k)\,dk, \\ \phi(T_B) - \phi(0) &= i\int_{k_0}^{k_0-2\pi} \langle w_k|\,\frac{\partial}{\partial k}\,|u_k\rangle\,dk, \end{aligned} \tag{19}$$

where T_B signifies a full Bloch period. From these equations it is clear that $\gamma(t)$ plays the role of a dynamic loss whereas $\phi(t)$ yields the Berry phase associated with the Bloch eigenstate $|u_k(x)\rangle$. In these equations, $\langle w_k|$ denotes the left Bloch eigenstate associated with $\hat{H}_{\text{eff},0}$. Note that here, thanks to the dissipative nature of the couplings, one can directly measure the Berry phase without any contamination from dynamical phases. This is in stark con-

trast to conventional Bloch oscillation techniques where conservative couplings inevitably mix the geometric phase with dynamical phases (21). From here, one can calculate the ratio of amplification/attenuation associated with Bloch eigenstates corresponding to different dissipative bands of a lattice. For instance, for a dissipative SSH model with dissipative intracell and intercell couplings Γ_A and Γ_B, this ratio is given by $\exp\left(2/v \int_{k_0}^{k_0-2\pi} E_+(k)\, dk\right)$, where $E_+(k) = \sqrt{\Gamma_A^2 + \Gamma_B^2 + 2\Gamma_A\Gamma_B}$.

Here, we would like to emphasize that although the Zak phases associated with the two configuations D_1 and D_2 shown in the Fig. 3 of the main text (i.e. ϕ_{Z1}, ϕ_{Z2}) depend on the choice of the unit cells in the lattice, the differential value $\phi_{Z1} - \phi_{Z2}$ remains invariant with respect to such transformations. To show this, assume that $|\psi(x)\rangle = \exp ikx\, |u_k(x)\rangle$ is the Bloch eigenstate associated with the SSH Hamiltonian (similar results apply to the effective Hamiltonian $\hat{H}_{\text{eff},0}$ associated with the dissipative SSH model). In this case, the Zak phase associated with this Bloch eigenstate is equal to $\phi_Z = \int_{-\pi/a}^{\pi/a} i\, \langle u_k| \frac{\partial}{\partial k} |u_k\rangle\, dk$. Under a shift in the coordinates $x' = x + d$, this Bloch eigenstate will transform to $\exp ikx \exp(-ikd)\, |u_k(x'-d)\rangle$. The new Zak phase will transform according to $\phi_Z' = \int_{-\pi/a}^{\pi/a} i\, \langle \exp(ikd) u_k| \frac{\partial}{\partial k} |\exp(-ikd) u_k\rangle\, dk$. From here, it is obvious that $\phi_Z' = \phi_Z + 2\pi/a \times d$. Therefore, although ϕ_{Z1} and ϕ_{Z2} can shift according to these relations, the differential Zak phase $\phi_Z = \phi_{Z1} - \phi_{Z2}$ remains invariant under the shift in the unit cells.

9 Gauge fields in multiple dissipative bands

In this section we extend the analysis of Section 5 to a multiband system describing the dissipative SSH model wherein transition among the bands is facilitated by dissipation. We start with the dissipative evolution equation $\partial/\partial t\, |\psi(x,t)\rangle = \hat{H}_{\text{eff,SSH}}\, |\psi(x,t)\rangle$ which supports Bloch eigenstates $|\psi_\pm(x,t)\rangle = \exp(ikx)\exp(-E_\pm(k)t)\, |u_{\pm,k}(x)\rangle$ associated with the upper (+) and lower (−) bands. Here, k is the Bloch momentum while $E_\pm(k)$ are real Bloch eigenvalues

that correspond to eigenstate amplification/decay in time. In addition, $|u_{\pm,k}(x)\rangle$ are the associated Bloch eigenstates within a unit cell characterized by the periodic boundary conditions $|u_{\pm,k}(x+a)\rangle = |u_{\pm,k}(x)\rangle$, with $a = 1$ being the size of a unit cell. Next, we consider a linearly increasing potential term in the evolution equation $\partial/\partial t\,|\psi(x,t)\rangle = \hat{H}_t\,|\psi(x,t)\rangle$, $\hat{H}_t = \hat{H}_{\text{eff,SSH}} + \hat{H}_1$, where $\hat{H}_1 = -i\alpha\hat{x}$, with α being the gradient of the potential which is proportional to the magnitude of the effective force. The instantaneous state of the system can then be given by the spinor $[a_+(t), a_-(t)]^T$ defined in such a way that $|\psi(x,t)\rangle = a_+(t)\exp[i(k_0 - vt)x]\,|u_{+,k_0-vt}(x)\rangle + a_-(t)\exp[i(k_0-vt)x]\,|u_{-,k_0-vt}(x)\rangle$. By substituting the latter in the evolution equation one would obtain

$$\begin{aligned}
&\frac{\partial a_+}{\partial t}\exp[i(k_0-vt)x]\,|u_{+,k_0-vt}(x)\rangle - ivxa_+\exp[i(k_0-vt)x]\,|u_{+,k_0-vt}(x)\rangle \\
&- va_+\exp[i(k_0-vt)x]\frac{\partial}{\partial k}\,|u_{+,k_0-vt}(x)\rangle + \frac{\partial a_-}{\partial t}\exp[i(k_0-vt)x]\,|u_{-,k_0-vt}(x)\rangle \\
&- ivxa_-\exp[i(k_0-vt)x]\,|u_{-,k_0-vt}(x)\rangle - va_-\exp[i(k_0-vt)x]\frac{\partial}{\partial k}\,|u_{-,k_0-vt}(x)\rangle = \\
&\left[E_+(k_0-vt) - i\frac{\alpha}{a}x\right]a_+(t)\exp[i(k_0-vt)x]\,|u_{+,k_0-vt}(x)\rangle + \\
&\left[E_-(k_0-vt) - i\frac{\alpha}{a}x\right]a_-(t)\exp[i(k_0-vt)x]\,|u_{-,k_0-vt}(x)\rangle\,.
\end{aligned} \tag{20}$$

From here, using the orthogonality of the upper/lower Bloch eigenstates it follows that $v = \alpha/a = \alpha$ and

$$\frac{\partial}{\partial k}\begin{pmatrix} a_+ \\ a_- \end{pmatrix} = \begin{pmatrix} \frac{-1}{v}E_+(k) - \langle w_{+,k}|\,\frac{\partial}{\partial k}\,|u_{+,k}\rangle & -\langle w_{+,k}|\,\frac{\partial}{\partial k}\,|u_{-,k}\rangle \\ -\langle w_{-,k}|\,\frac{\partial}{\partial k}\,|u_{+,k}\rangle & \frac{-1}{v}E_-(k) - \langle w_{-,k}|\,\frac{\partial}{\partial k}\,|u_{-,k}\rangle \end{pmatrix}\begin{pmatrix} a_+ \\ a_- \end{pmatrix}, \tag{21}$$

where $\langle w_{\pm,k}|$ denote the upper/lower left Bloch eigenstates associated with $\hat{H}_{\text{eff,SSH}}$.

In the more general case of higher-dimensional lattices, Eq.21 takes the form

$$\frac{\partial}{\partial t}\begin{pmatrix} a_+ \\ a_- \end{pmatrix} = \begin{pmatrix} E_+(k) + i\mathbf{A}_{++}\cdot\boldsymbol{F} & i\mathbf{A}_{+-}\cdot\boldsymbol{F} \\ i\mathbf{A}_{-+}\cdot\boldsymbol{F} & E_-(k) + i\mathbf{A}_{--}\cdot\boldsymbol{F} \end{pmatrix}\begin{pmatrix} a_+ \\ a_- \end{pmatrix}. \tag{22}$$

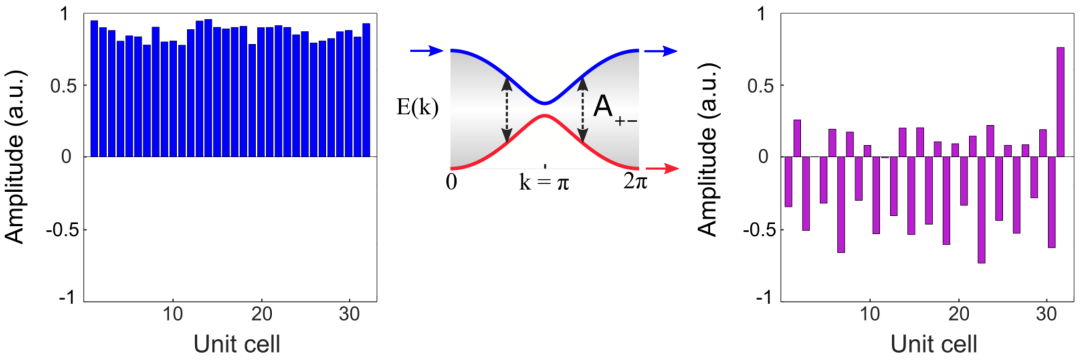

Figure 5: **Tunneling between the Bloch bands of a dissipative SSH model.** Experimental data representing how the Bloch eigenstates from the upper band tunnel to the lower band at the end of one Bloch period.

This immediately yields Eq. 2 in the main text.

Equation 22 predicts a tunneling rate proportional to the off-diagonal elements of the Wilczek-Zee matrix defined in the main text. As a result, at the end of a Bloch period, the output of our dissipatively coupled time-multiplexed resonator network is characterized by a mixture of the upper and lower bands. This is evident in the measurement results presented in Fig. 5 corresponding to a scenario when the upper band is initially excited.

In addition, Eq. 22 also accounts for the difference in the geometric phases exhibited by the upper/lower bands of the dissipative SSH model. Figure 6 shows theoretically predicted results for a wide range of the effective force $\left|\overrightarrow{F}\right|$ relative to the bandgap width. These values are clearly consistent with our experimentally measured values reported in the main text.

As mentioned in the main text, the reason behind different geometric phases associated with the upper and lower bands of the dissipative SSH model is the relative amplification/attenuation experienced by their corresponding Bloch eigenstates. To further clarify this, we analyzed the Bloch dynamics associated with the band structures of both the conservative and the dissipative SSH models governed by the modified Wilson line operator. Figures 7 and 8 show the obtained results. In the conservative case, when either of the upper/lower bands are excited at the input,

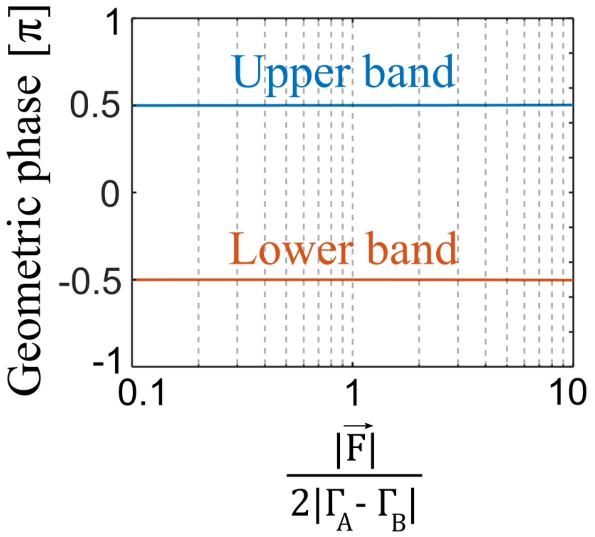

Figure 6: **Geometric phases associated with the upper/lower bands of the dissipative SSH model.** Theoretically predicted values for the geometric phases associated with the two bands of the dissipative SSH model vs. the magnitude of the applied effective force normalized to the width of the bandgap.

by the end of the Bloch period the output wavefunction predominantly remains in the same Bloch band (within the adiabatic conditions), hence resulting in identical geometric phases. In contrast, when the lower Bloch band in a dissipative SSH model is excited in the beginning, at some point along the Bloch period its associated wave amplitude tends to vanish due to attenuation (Fig. 8b). Meanwhile, the upper-band Bloch eigenstate which is generated via interband transitions tends to grow. Therefore, once the lower-band eigenstates are repopulated by the upper-band, their associated geometric phase follows that associated with the upper one, except for an added π phase shift created due to successive tunnelings (Fig. 8b). Remarkably, as evident from these figures, this picture does not qualitatively change as one alters the strength of the applied effective force responsible for BO.

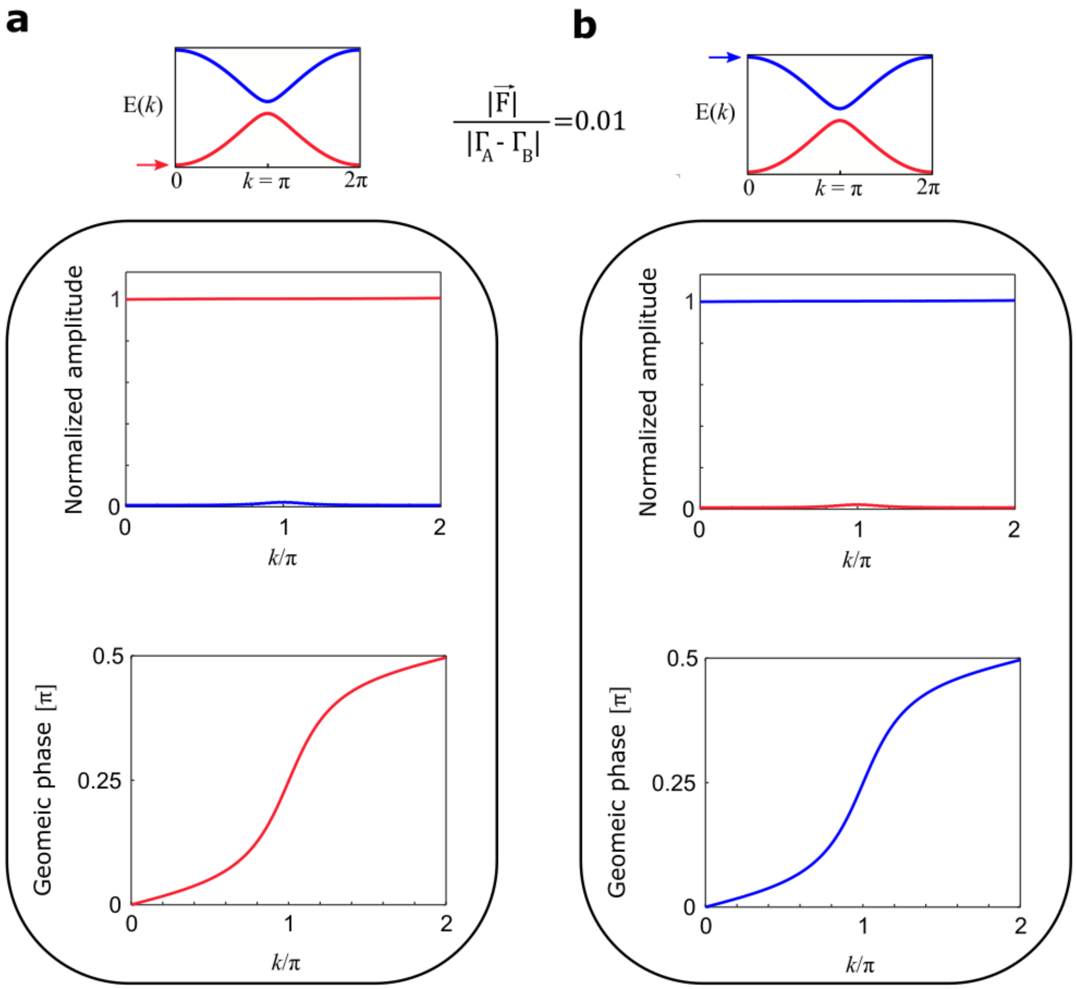

Figure 7: **Geometric phases in a conservative SSH model.** Theoretically predicted geometric phases as well as normalized Bloch eigenstate amplitudes associated with the **a,** upper and **b,** lower bands in a conservatively coupled SSH model in the adiabatic regime.

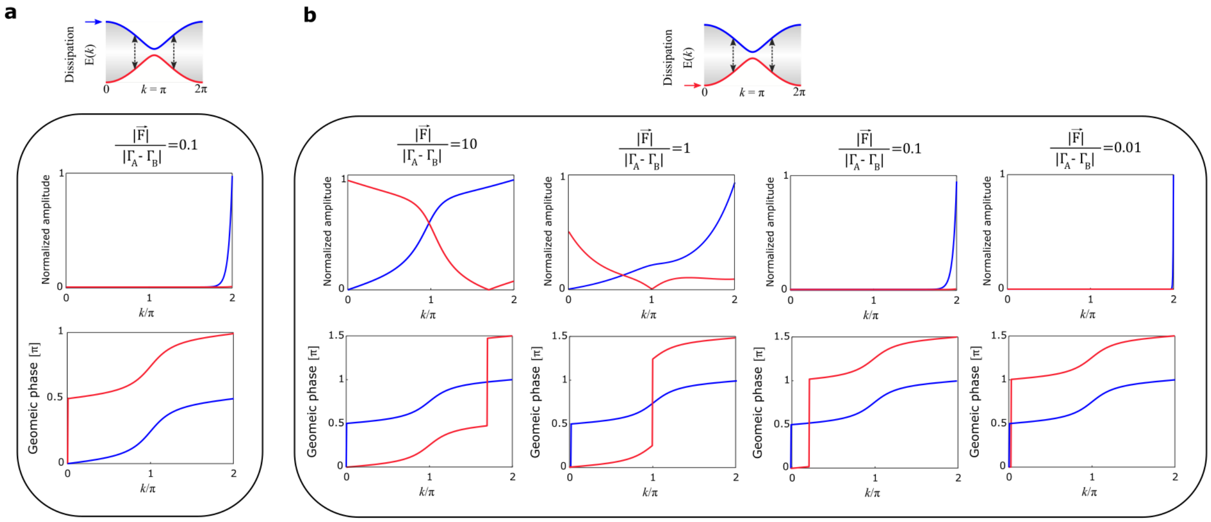

Figure 8: **Geometric phases in a dissipative SSH model.** Theoretically predicted geometric phases as well as normalized Bloch eigenstate amplitudes associated with the **a,** upper and **b,** lower bands in a dissipatively coupled SSH model for different values of the applied effective force normalized to the coupling strengths.

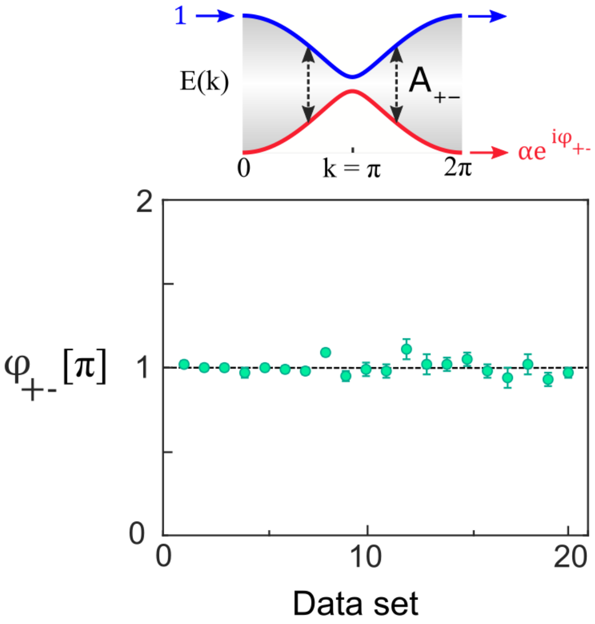

Figure 9: **Combined tunneling and geometric phases in the dissipative SSH model.** Experimentally measured values associated with the combined geometric and tunneling phases acquired by the Bloch eigenstates as they tunnel from the upper to the lower band in one Bloch period.

Finally, Eq. 22 predicts a phase accumulation of π by the Bloch eigenstates as they transition between the bands over one Bloch period. To confirm this experimentally, we initially excited the upper band in the network and monitored the phase associated with the lower-band Bloch eigenstate at the end of one complete period. Figure 9 shows experimentally measured values that corroborate the aforementioned theoretical results.

10 Dissipative honeycomb lattice

For the dissipatively coupled honeycomb lattice with zero detuning between its sublattices that is considered in the main text, the effective Hamiltonian in the reciprocal space becomes

$$\hat{H}_{\text{eff,HC}}(\boldsymbol{k}) = \begin{pmatrix} 0 & t(\boldsymbol{k}) \\ t^*(\boldsymbol{k}) & 0 \end{pmatrix}, \tag{23}$$

where $t(\boldsymbol{k}) = |t(\boldsymbol{k})|e^{i\theta(\boldsymbol{k})} = \kappa(e^{i\boldsymbol{k}.\boldsymbol{d}_1} + e^{i\boldsymbol{k}.\boldsymbol{d}_2} + e^{i\boldsymbol{k}.\boldsymbol{d}_3})$. From here, it is easy to show that

$$\hat{\mathbf{A}} = 1/2 \begin{pmatrix} \boldsymbol{\nabla}_{\boldsymbol{k}}\theta(\boldsymbol{k}) & -\boldsymbol{\nabla}_{\boldsymbol{k}}\theta(\boldsymbol{k}) \\ -\boldsymbol{\nabla}_{\boldsymbol{k}}\theta(\boldsymbol{k}) & \boldsymbol{\nabla}_{\boldsymbol{k}}\theta(\boldsymbol{k}) \end{pmatrix}. \tag{24}$$

Figure 5 of the main text shows results based on Eq. 22 and 24 when $|\boldsymbol{F}| = \kappa$ for different directions of the force $\boldsymbol{F}$ aligned with the paths C_1 and C_2, as shown in the main text.

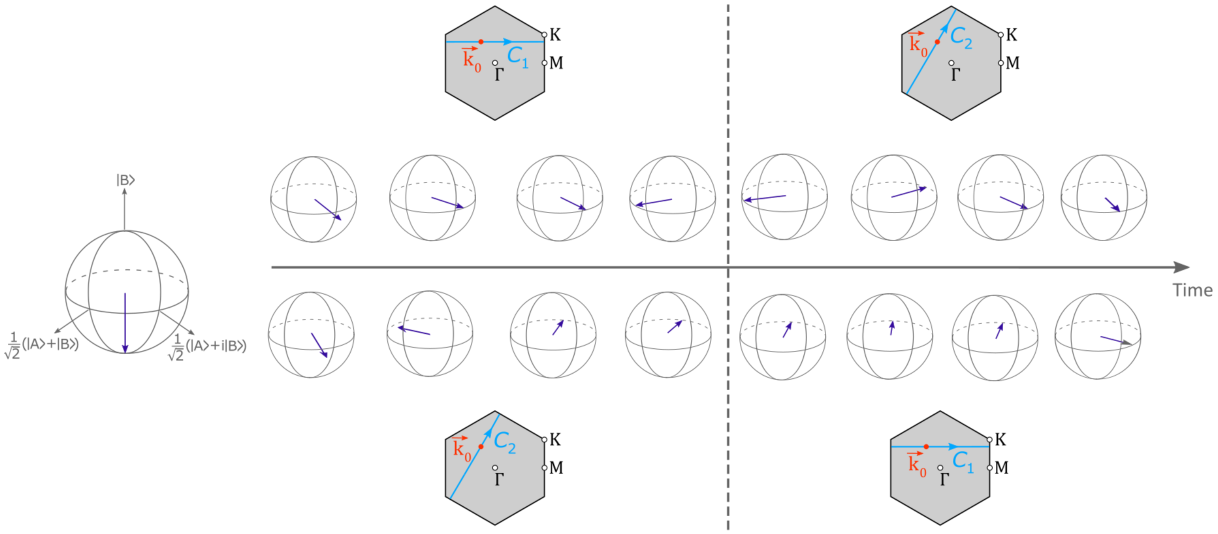

Figure 10: **Non-Abelian dynamics in a dissipative honeycomb lattice.** Simulation results showing the evolution of states on the Bloch sphere in a dissipative honeycomb lattice. It is evident that different operators corresponding to momentum-space dynamics along different closed loops C_1 and C_2 within the Brillouin zone do not commute and display non-Abelian behavior.

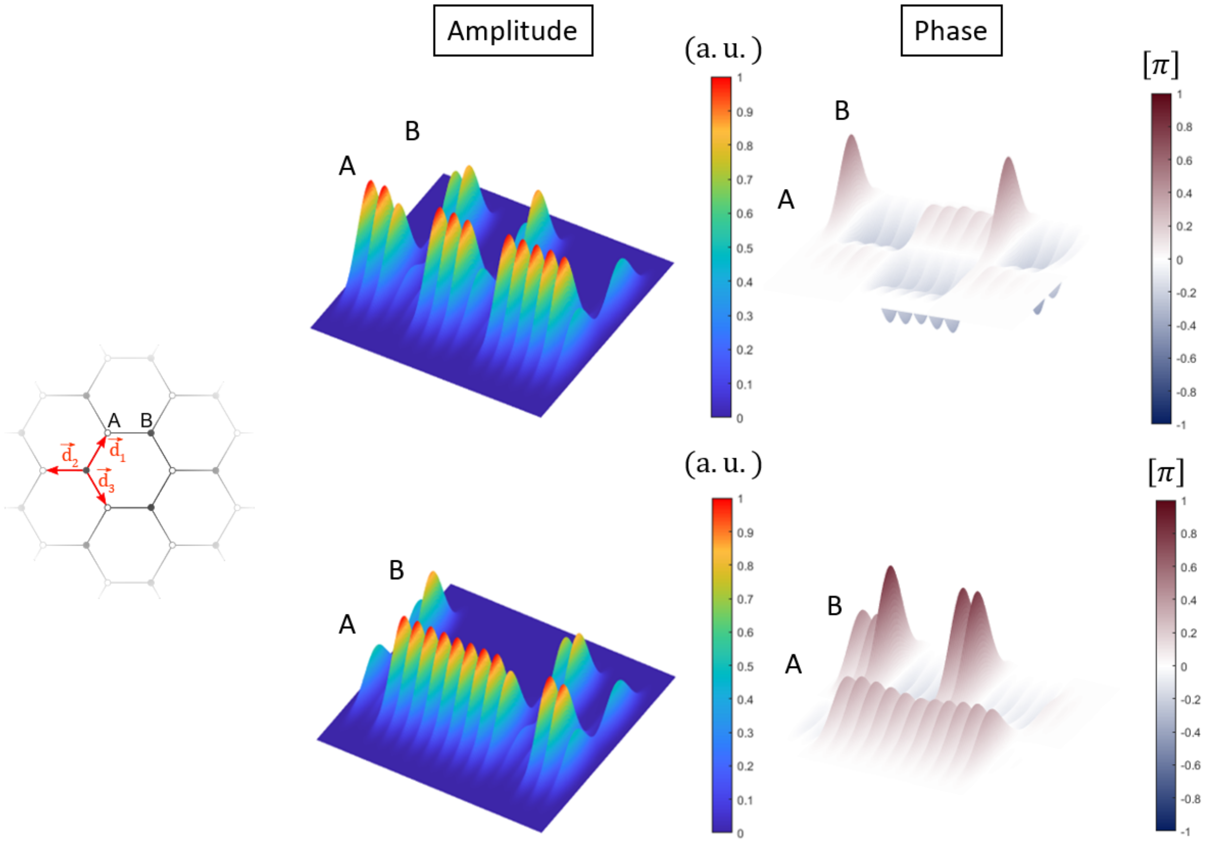

Figure 11: **Non-Abelian state evolutions in a dissipative honeycomb lattice.** Simulation results regarding discrete state evolutions under different momentum-space operators correpsonding to different closed-loops C_1 and C_2 within the Brillouin zone in a dissipative honeycomb lattice. The plots show amplitudes and phases of the fields associated with the A and B sites within a unit cell of the lattice (shown on the left panel).

11 Possible Implementation of a Honeycomb Lattice in a Time-Multiplexed Resonator Network

In Fig. 12a, we present the schematic of a time-multiplexed network capable of implementing a dissipatively coupled honeycomb lattice with PBCs along both directions. This network maps to the honeycomb lattice shown in Fig. 12b with N_x sites along the vertical direction and N_y sites along the horizontal direction. For concreteness, we have chosen $N_x = 4$ and $N_y = 10$

in the figure. The coloration of the delay lines in Fig. 12a corresponds to the coloration of the couplings in Fig. 12b.

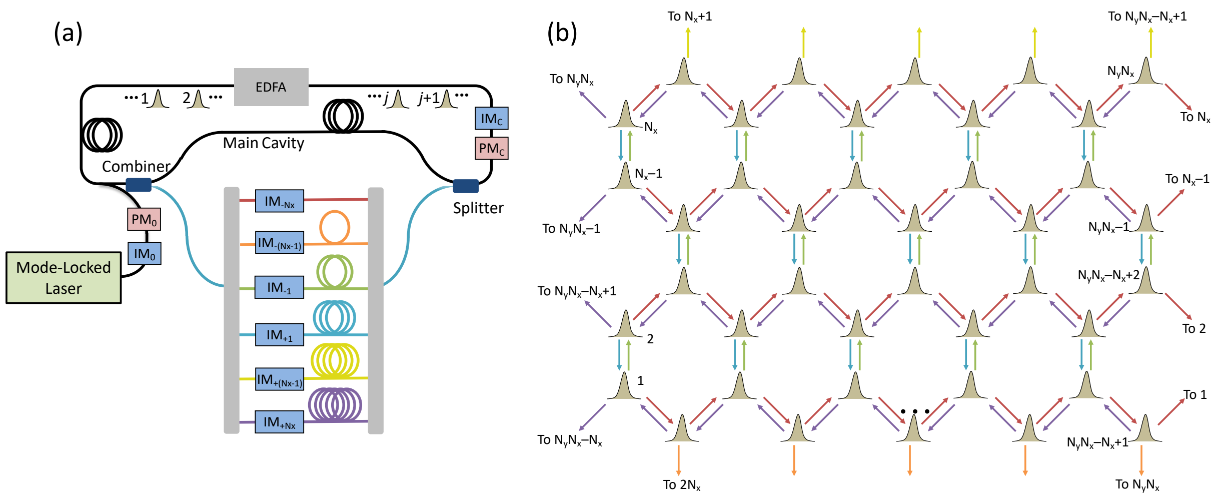

Figure 12: **Possible Implementation of a Honeycomb Lattice with a Time-Multiplexed Resonator Network.** **a**, A time-multiplexed network with six delay lines, which can be used to implement a dissipatively coupled honeycomb lattice with PBCs along both directions. **b**, The mapping between the network in **a** and a honeycomb lattice. The coloration of the couplings corresponds to the coloration of the delay lines.
